# Microanatomy and Development of the Dwarf Male of *Symbion pandora* (Phylum Cycliophora): New Insights from Ultrastructural Investigation Based on Serial Section Electron Microscopy

**DOI:** 10.1371/journal.pone.0122364

**Published:** 2015-04-15

**Authors:** Ricardo Cardoso Neves, Heinrich Reichert

**Affiliations:** Biozentrum, University of Basel, Klingelbergstrasse 50, CH-4056, Basel, Switzerland; University of Würzburg, GERMANY

## Abstract

Cycliophorans have a complex life cycle that involves several sexual and asexual stages. One of the sexual stages is the 40 μm-long dwarf male, which is among the smallest free-living metazoans. Although the dwarf male has a highly complex body plan, this minute organism is composed of a very low number of somatic cells (~50). The developmental processes that give rise to this unique phenotype are largely unknown. Here we use high resolution serial block face—scanning electron microscopy to analyze the anatomy and morphogenesis of three cycliophoran dwarf males at different developmental stages ranging from internal bud to mature male. The anatomical and morphological features of the mature dwarf male stage reported here largely correspond to those reported in earlier studies. Interestingly, the organs that typically characterize the anatomy of the mature dwarf male, e.g., muscles, brain, testis and glands, are already formed in the young male. However, there are striking differences between the mature male and young male stages at the level of cellular architecture. Thus, while the young male stage, like the internal bud stage, possesses approximately 200 nucleated cells, the mature male stage comprises only around 50 nucleated cells; muscle and epidermal cells of the mature male lack nuclei. Moreover, the total body volume of the mature male is only 63% of the body of the young male implying that the maturation of the young male into a mature male involves a marked reduction of internal body volume, mainly by massive nuclei loss. Our comparative analysis of these dwarf male specimens reveals unprecedented insight into the striking morphological and developmental differences that characterize these highly miniaturized male stages both at the level of body organization and at the level of cellular ultrastructure.

## Introduction

The phylum Cycliophora consists of microscopic, commensal invertebrates living epizoically on the mouthparts of a small number of nephropid decapod species [1, 2]. Only two cycliophoran species have been described so far; *Symbion pandora*, Funch and Kristensen, 1995 is found on the Norway lobster, *Nephrops norvegicus* Linneus 1758, while *Symbion americanus* Obst, Funch and Kristensen, 2006 lives on the American lobster, *Homarus americanus* H. Milne-Edwards, 1837. In addition, an undescribed cycliophoran species is also an epizoite on the European lobster, *Homarus gammarus* Linnaeus, 1758 [3]. Moreover, evidence from the molecular characterization of cycliophorans suggests the existence of cryptic species in *S*. *americanus* [4–6]. Recently, the first evidence was provided for a symbiotic relationship between cycliophorans and organisms other than nephropid lobsters, namely harpacticoid copepods [7].

**Fig 1:**
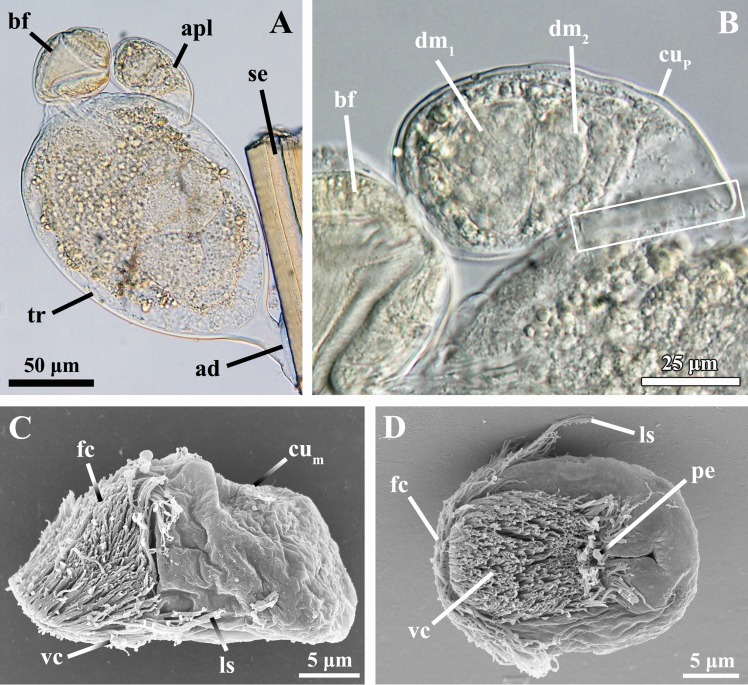
The feeding stage, attached Prometheus larva and dwarf male of *Symbion pandora*. Light (A, B) and scanning electron micrographs (C, D). Anterior faces left in C and D. **A:** Feeding stage with attached Prometheus larva (apl). The closed buccal funnel (bf) is facing upwards. **B:** Close up of the attached Prometheus larva. Note the two dwarf males (dm_1–2_) inside the larval body. The rectangle outlines the region of interface between the cuticle of the attached Prometheus larva and that of the feeding stage. **C:** Dwarf male, lateral view. The frontal ciliated field (fc) extends dorsally, covering the antero-dorsal region of the body. **D:** Dwarf male, ventral view. The ventral ciliated field (vc) spans from the most anterior body region until the penial organ (pe). Abbreviations: ad adhesive disk, cu_m_ cuticle of the dwarf male, cu_P_ cuticle of the attached Prometheus larva, ls lateral sensory organ (sensilla), se host’s seta, tr trunk.

Cycliophorans have a highly complex life cycle that involves various sexual and asexual stages [8, 9]. The most prominent stage is a sessile, feeding individual (Fig. 1A), which is characterized by a body divided into an anterior ciliated buccal funnel, an oval trunk, and a posterior stalk ending as an attachment disk [1]. A single feeding individual is able to asexually generate free swimming stages, one at a time, by a budding process that occurs inside the trunk [2]. Among these is the Pandora larva, a free swimming larval stage. Once freed, the Pandora larva settles close to the maternal feeding individual and develops into a new feeding stage by protruding an internally generated buccal funnel. Also generated inside the feeding stage, but involved in the sexual phase of the life cycle, are the Prometheus larva and the female. Once released, the Prometheus larva settles head-down on the trunk of a feeding individual (Fig. 1A), usually close to the buccal funnel, and generates one to three dwarf males inside its body (Fig. 1B) [9, 10]. Each of these dwarf males develops inside the Prometheus larva from an internal bud-like cluster of cells. While almost all the internal (and external) structures of the larva degenerate [9], several muscle fibers of the larva remain surrounding the dwarf males, which thus appear to have a protective role [10]. The female, carrying inside a single large oocyte, is impregnated by the dwarf male and the embryo develops into a chordoid larva, which subsequently settles on a new host individual and eventually develops into a new feeding stage [11, 12]. However, many aspects of the life cycle remain elusive. For instance, it is still unclear when and where the male impregnates the female. Although a breeding event has been suggested to occur after the liberation of the female and the dwarf male from inside the feeding stage and the Prometheus larva, respectively, this process has never been observed [8, 9].

With a body length of about 40 μm, the cycliophoran dwarf male (Fig. 1C,D) is among the smallest free-living metazoans. The dwarf male exhibits a complex bodyplan [10, 13, 14]. Its external morphology is characterized by dense ventral and frontal ciliated fields, sensorial tufts of cilia (sensilla) situated laterally and frontally, and a penis located ventro-posteriorly inside a cuticular pouch-like structure [9, 15]. In addition, frontal palps and a dorsal papilla are other sensorial elements present [9, 15, 16]. The actual function of the penis as a true copulatory organ or as a mere anchoring and piercing organ is still under debate [12, 15]. Based on the latter interpretation, the impregnation of the female by hypodermic transfer of sperm even before its liberation from the feeding stage has also been suggested [8].

The internal anatomy of the dwarf male has been characterized by transmission electron and confocal laser scanning microscopy [9, 10, 13–15]. A complex muscular architecture has been described and includes several dorsoventral muscles and longitudinal muscles that span the body dorsally and ventrally [10, 13]. In the nervous system, an anterior, relatively large brain—described as occupying about one third of the body volume—is composed of a dorsolateral pair of ganglia interconnected by a commissural neuropil [9, 17]. Both ganglia consist of a cluster of perikarya that are characterized by large nuclei, uniformly distributed heterochromatin and few mitochondria. A pair of longitudinal nerves project latero-ventrally from the posterior region of the brain to the base of the penis. Several glands are present throughout the cycliophoran male body. Three pairs of so-called cerebral glands are located anteriorly, close to the brain. The cytoplasmic necks of all of these unicellular cerebral glands extend ventrally around the neuropil and descend towards the antero-ventral region, where they open with separated outlets. Also located close to the brain, a pair of unicellular medial glands extends from the area between the cerebral ganglia to the ventro-posterior region of the male body, where they open with separated outlets as well. Moreover, two pairs of so-called prostate glands are associated with the penial organ. Because the outlets of these glands appear to be absent, it has been suggested that their content is secreted either with the sperm or into the penis lumen [9]. Finally, the dwarf male possesses a single testis located in the posterior region of the body, dorsally to the penial organ.

The complex body plan of the minute, free-living cycliophoran male has attracted considerable interest because of the highly reduced number of somatic cells present in these organisms. An investigation based on confocal and transmission electron microscopy has shown that the sexually mature dwarf male of *Symbion pandora* and *S*. *americanus* possesses, on average, only 52 and 59 somatic cells, respectively [14]. Interestingly, however, in some individuals of *S*. *pandora* up to 84 somatic cells were found, and in some of the investigated males of *S*. *americanus* well over 100 somatic cells, namely 106, 142 and 170, were observed. Whether this discrepancy is related to differences in the developmental stadium of the investigated dwarf males is an open question. Indeed, the developmental biology of the dwarf male—or any other cycliophoran life cycle stage—currently remains unknown.

In this report, we use serial block-face scanning electron microscopy (SBF-SEM) to reconstruct the internal anatomy of the cycliophoran dwarf male. Based on SBF-SEM data, we analyse all of the cells that comprise the dwarf male at a 3D single-cell level. Moreover, since our ultrastructural study is focused on a Prometheus larva that contains two differentiated dwarf males at different developmental stages, we provide detailed anatomical information both on a fully developed, mature adult and on a juvenile, young dwarf male. In addition, in the same Prometheus larva, we characterize the cellular features of a third, largely undifferentiated bud-like stage from which a differentiated dwarf male could eventually develop. Our comparative analysis of these dwarf male specimens reveals unprecedented insight into the striking morphological and developmental differences that characterize these stages both at the level of body organization and at the level of cellular ultrastructure.

## Material and Methods

### Collection and fixation of specimens

Specimens of the Norway lobster, *Nephrops norvegicus*, were caught with cages by a local fisherman in the Gulmarsfjorden, Sweden (58°15’N, 11°26’W), in June 2012 and retained for several days in running seawater without any food supply. Mouthparts from one of the lobsters were dissected from the host and placed in Petri dishes with filtered seawater. Feeding stages of *Symbion pandora* with attached Prometheus larvae were gently shaved from the host mouthparts using a scalpel, and fixed with 2.5% glutaraldehyde in 0.2 M sodium cacodylate (pH = 7,2) at room temperature (RT). The use of Norway lobsters and Cycliophora in the laboratory does not raise any ethical issues and therefore Regional or Local Research Ethics Committee approvals are not required. Moreover, no specific permits were required for the described field studies, as none of the investigated species are included in any endangered list, at national or international levels.

### Serial block face scanning electron microscopy

#### Sample preparation

Fixed specimens were rinsed in 2.5% glutaraldehyde, 2% formaldehyde in 0.2 M sodium cacodylate (pH = 7,2) for 2–3 hours on ice. Afterwards, specimens were prepared using a protocol as described in [18]. Samples were washed 5×3 minutes in cold 1M cacodylate buffer containing 2 mM calcium chloride and then post-fixed in a solution containing 1.5% potassium ferrocyanide and 2% osmium tetroxide in 0.1 M cacodylate buffer with 2 mM calcium chloride for 1 hour on ice. The specimens were then washed in double distilled water (ddH_2_O) 5×3 minutes at RT and stained in a 1% thiocarbohydrazide solution in ddH_2_O for 20 minutes at RT. Afterwards, specimens are washed in ddH_2_O 5×3 minutes and rinsed in 2% aqueous osmiumtetroxide for 30 minutes at RT. Following this staining, specimens were washed in ddH_2_O 5×3 minutes, at RT, and then stained in 1% uranyl acetate in ddH_2_O at 4°C, overnight. The day after, specimens were washed in ddH_2_O 5×3 minutes, at RT, and then rinsed in a pre-warmed Walton’s lead aspartate solution (0.066 g of lead nitrate dissolved in 10 ml of 0.03 M aspartic acid stock and pH adjusted to 5.5 with 1N HOK) for 30 minutes, at 60°C. After 5×3 minutes washing in ddH_2_O at RT, samples were dehydrated through an ethanol series and transferred to acetone. Finally, the samples were embedded in Durcupan resin (TAAB Laboratories Equipment Ltd), which was polymerized at 60°C for 2 days according to the manufacturer’s instructions.

#### Acquisition of image stacks

The sample selected to investigate was trimmed to expose the Prometheus larva on all their surfaces and the block was mounted on aluminium specimen pins (Gatan Inc.) using cyanoacrylate glue. The block was further trimmed with a glass knife to as small a size as possible, so that the tissue was exposed from all sides. Subsequently, the entire surface of the specimen was sputter coated with a thin layer of a gold/palladium alloy and inserted into the 3View microtome (Gatan Inc.), in the chamber of a Quanta 200 VP-FEG (FEI Company). The image stack was acquired by performing serial SEM of the block face. A section 80 nm thick was cut from the block surface with a diamond knife and the freshly cut surface of the block was scanned; this process was repeated sequentially to collect 676 slices over 4 days. This image acquisition cycle was repeated until the Prometheus larva had been completely imaged. The electron microscope was operated at an acceleration voltage of 2.5 kV, with a standard 30 μm aperture in low vacuum mode (0.12Torr). All images were taken with the following scanning settings: dwell time = 7 μs per pixel; image size = 4096 × 4096 pixels (pixel size = 43 nm).

#### Stack alignment and 3D imaging

All of the images for a single attached Prometheus larva were imported into a single stack that was automatically aligned using the TrakEM2 plugin in a Fiji/ImageJ software package (http://fiji.sc/Fiji). Individual subcellular and organelle structures were manually segmented by using TrakEM2: the dissector tool was used to count cells, while the area list tool was used to tag regions of interest with different colours and render 3D imaging reconstructions.

## Results

**Fig 2:**
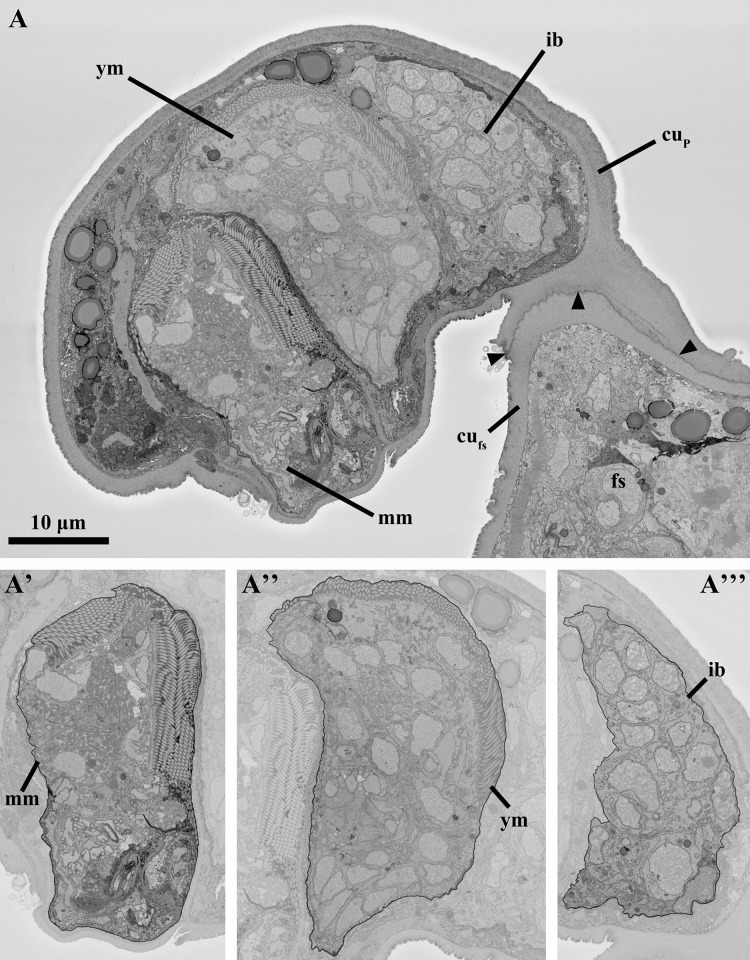
Anatomy of the attached Prometheus larva of *Symbion pandora*. SEM-SBF micrographs, longitudinal sections. Anterior is up and ventral is right in A’ and A”. (**A**) Prometheus larva settled on the trunk of a feeding stage (fs). Note the interface (arrowheads) between the attached Prometheus larva and the feeding stage. (A’) Mature male (mm). (A”) Young male (ym). (A”’) Internal bud (ib). Abbreviations: cu_fs_ cuticle of the feeding stage, cu_P_ cuticle of the attached Prometheus larva.

The attached Prometheus larva investigated contains two differentiated dwarf males, one at a mature stage and a second at young stage, as well as a third largely undifferentiated bud-like stage (Fig. 2A, S1 Video). Both the larva and the dwarf males are sectioned longitudinally; the main body axis of the dwarf males is orthogonal to the main body axis of the larva. The two dwarf males have the same orientation; their ventral region is oriented towards the anterior region of the Prometheus larva that is attached to the feeding stage. The dwarf male located posteriorly in the larval body appears to be fully differentiated and mature (Fig. 2A’). The second dwarf male, which occupies a middle position inside the larva, appears to correspond to a younger stage that has not completed its development (Fig. 2A”). Finally, the third largely undifferentiated stage located anteriorly in the larval body region i.e., close to the contact site with the feeding stage, most probably corresponds to an internal bud from which a third dwarf male would be produced (Fig. 2A’’’). Thus, our ultrastructural analysis of a single attached Prometheus larva provides information on the anatomy and morphogenesis of the cycliophoran dwarf male at three different developmental stages, from internal bud to mature male.

### The mature dwarf male stage

In general, the anatomical features of the mature male specimen are in accordance with earlier descriptions of the cycliophoran dwarf male (see [9, 15]). The body of the mature male comprises only 47 nucleated cells (see Table 1). The majority of the cells with nuclei present in the mature male are those of the nervous system, which are in total 34 cells (ca. 72% of total nucleated cells). Eight nucleated cells (17%) correspond to the six cerebral and the two medial gland cells. Only three cells with nuclei (6.4%) appear to be epithelial or mesenchymal, while two nucleated cells (4.3%) are putatively related to the gonads. The nuclei of the muscle cells are absent at this stage, and the myocytes are composed mainly of muscle fibers surrounded by a small volume of cytoplasm containing mitochondria. The maximum length, height and width of the mature male is 27 μm, 12 μm and 28 μm, respectively, which means a body volume of approximately 9000 μm^3^ (see Table 2).

**Table 1:** Comparison of the number of nucleated cells (and respective percentage) composing the internal bud, young male and mature male.

	**Internal bud**	**Young male**	**Mature male**
Epithelium/Mesenchyme	-	114 (55.00%)	3 (6.40%)*
Nervous system	-	49 (23.60%)	34 (72.00%)
Musculature	-	32 (15.40%)	0 (0.00%)
Secretory glands	-	8 (3.85%)	8 (17.00%)
Testis	-	4 (1.90%)	2 (4.25%)
Prostate glands	-	1 (0.48%)	nf
**Total**	**186**	**208**	**47**

Details on the number of cells composing the organ systems are not applicable for the internal bud.

*this number corresponds to the solely three nuclei interpreted as belonging to epithelial/mesenchymal cells though the epithelium in the mature male is reduced to a thin layer of enucleated cells;-, not applicable; nf, not found.

**Table 2:** Comparison of the size (μm) of various anatomical aspects between the internal bud, young male and mature male.

	**Internal bud**	**Young male**	**Mature male**
Body:			
length	29	30	27
height	15	14	12
width	32	34	28
[internal volume (μm^3^)]	[13900]	[14300]	[9000]*
Maximum and minimum diameter of the neuropil middle region	-	3.85 and 2.80	4.70 and 2.50
Penis:			
base	-	2.8	3.3
tip	-	1.1	1.3
cuticle thickness	-	0.06	0.25
Maximum diameter of testis	-	8.8	7.8
Sperm cell:			
head length	-	8.5	8.5–10.5
head width	-	0.9	1.3–2.0
Diameter of the ventral glands	-	2.3	2.80
Diameter of the medial gland cells	-	3.2	2.9
Diameter of the cerebral gland cells	-	3.5–3.7	3.0–3.5

For the internal bud, only details on the body volume are applicable.

*Note that this value represents only ca. 63% of the internal volume of the young male body;-, not applicable.

**Fig 3:**
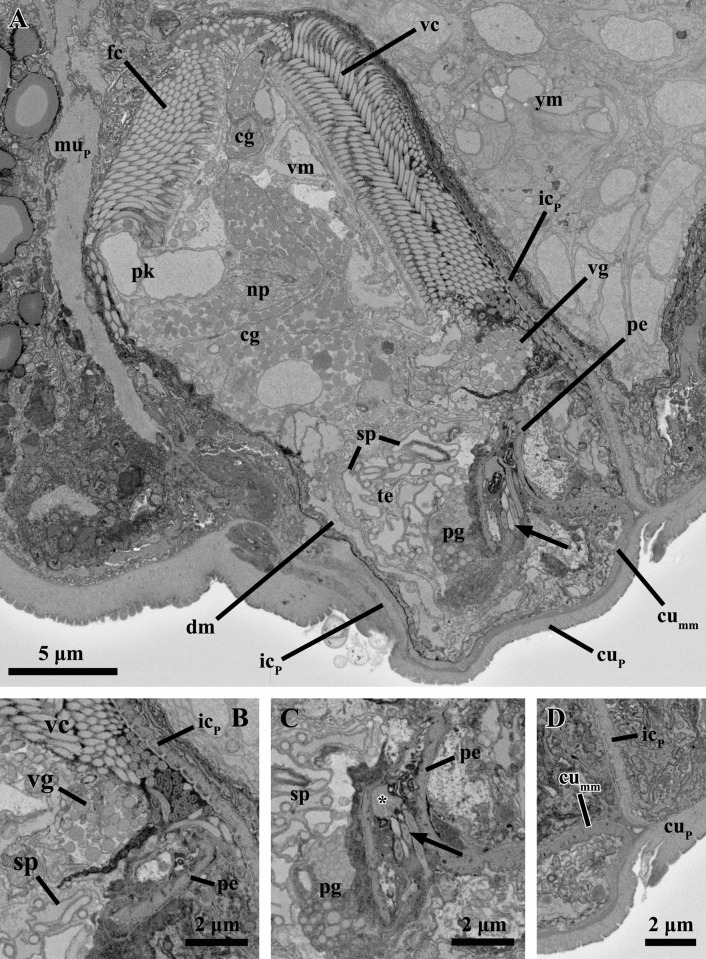
Anatomy of the mature male inside the attached Prometheus larva of *Symbion pandora*. SEM-SBF micrographs, longitudinal section. (**A**) Mature male surrounded antero-dorsally by a larval muscle fiber (mu_P_) and ventrally by an internal layer of larval cuticle (ic_P_). The young male (ym) is situated ventral to the mature male. Frontal (fc) and ventral ciliated fields (vc) characterize the external morphology of mature male. The penis (pe) and the single testis (te) are located posteriorly inside the mature male body. Note also the perikarya (pk) and the neuropil (np) that compose the brain, as well as the cerebral glands (cg) located anteriorly. (**B**) A single ventral gland (vg) is located close to the penis tip. **(C)** Close up of the penis with a sperm cell inside. The asterisk marks the nucleus, while the arrow points to the cilium. **(D)** Detail of the layer of internal cuticle deriving from the external cuticle of the larva (cu_P_). Abbreviations: cu_mm_ cuticle of the mature male, pg prostate glands, sp sperm cells, vm ventral muscle.

**Fig 4:**
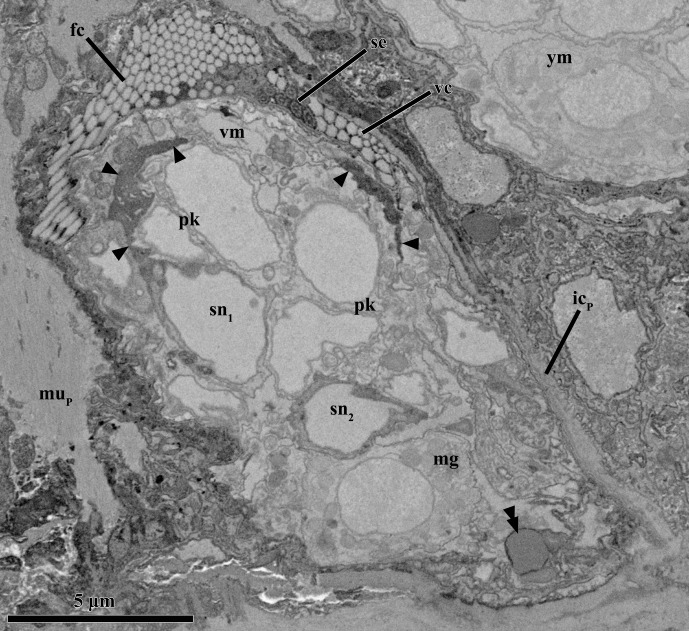
Anatomy of the mature male inside the attached Prometheus larva of *Symbion pandora*. SEM-SBF micrographs, longitudinal section. Two bipolar sensory neurons (sn_1_ and sn_2_) project cell processes (arrowheads), into the anterior sensilla (se). Note also the medial gland (mg), as well as the large vacuoles posteriorly (double arrowheads). Abbreviations: cu_mm_ cuticle of the mature male, fc frontal ciliated field, ic_P_ internal layer of larval cuticle, mu_P_ larval muscle fiber, pg prostate glands, pk perikarya, vc ventral ciliated field, vm ventral muscle, ym young male.

**Fig 5:**
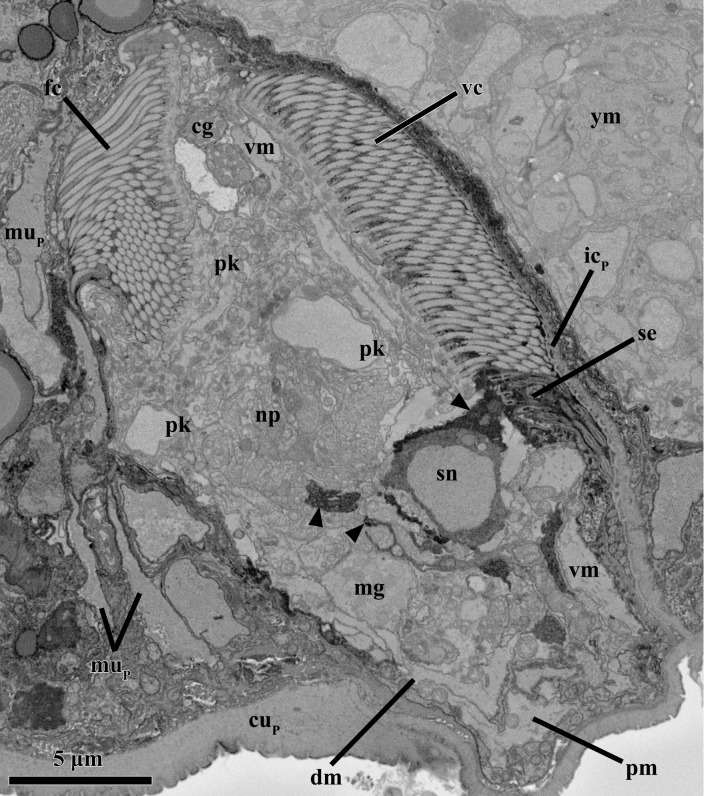
Anatomy of the mature male inside the attached Prometheus larva of *Symbion pandora*. SEM-SBF micrographs, longitudinal section. A bipolar sensory neuron (sn) projects cell processes (arrowheads) into the neuropil (np) as well as into the sensilla (se) located ventrally. Note the several muscles spanning ventrally (vm), dorsally (dm) and posteriorly (pm). Abbreviations: cg cerebral glands, fc frontal ciliated field, ic_P_ internal layer of larval cuticle, mg medial gland, mu_P_ larval muscle fiber, pk perikarya, vc ventral ciliated field, ym young male.

**Fig 6:**
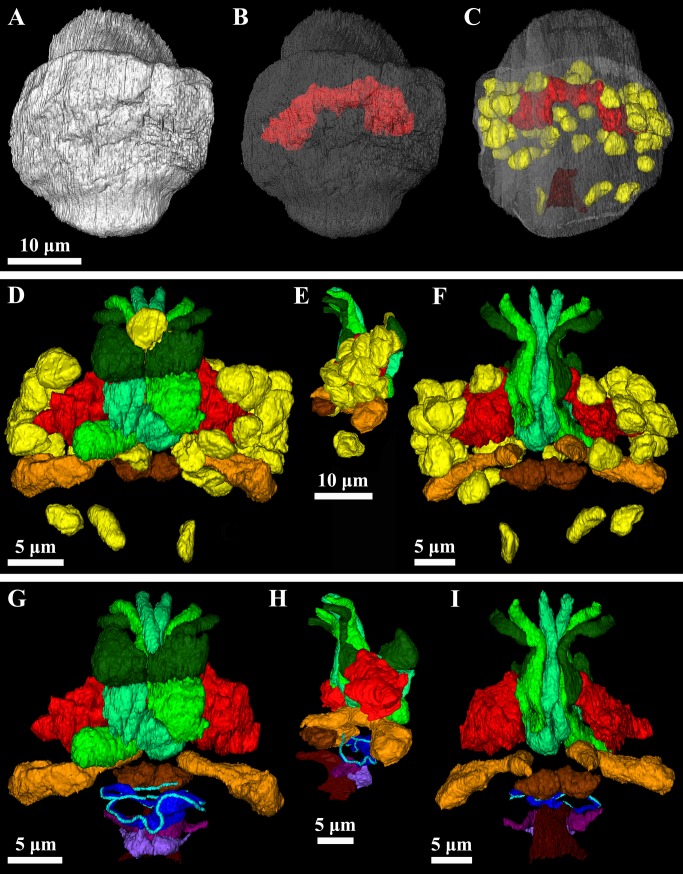
Three-dimensional reconstructions of various anatomical aspects of the mature dwarf male. *Symbion pandora*. (**A**) Outline of the cuticle (white), dorsal view. Note that the anterior ciliated field is not included. (**B**) Dorsal view of the neuropil (red) through the transparent outline of the cuticle as seen in A. **(C)** Ventral view of the brain, somatic nuclei (yellow) and penis (grenat) through the transparent outline of the cuticle as seen in A. Note the relative position of the neuropil and somatic nuclei inside the body. **(D-F)** Visualization of the cerebral glands (different shades of green), medial glands (orange) and ventral glands (brown). The neuropil and somatic nuclei are also included. **(D)** Dorsal view. **(E)** Lateral view. **(F)** Ventral view. **(G-I)** Visualization of the prostate glands (S-shaped are violet, while drop-shaped are purple) and sperm cells (heads are dark blue, while cilia are light blue). The penis, cerebral glands, medial glands, ventral glands and neuropil are also included. **(G)** Dorsal view. **(H)** Lateral view. **(I)** Ventral view.

**Fig 7:**
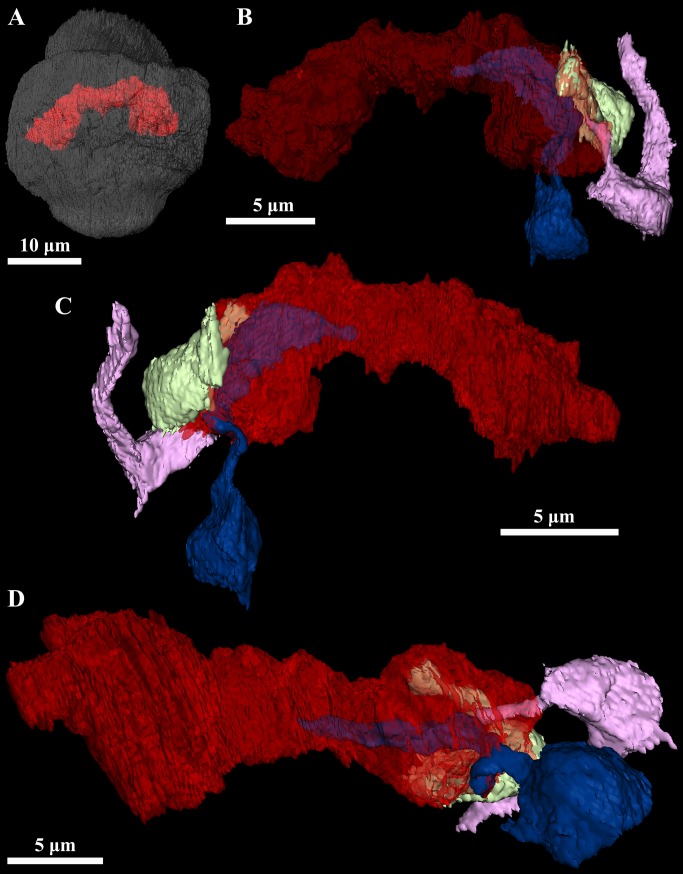
Three-dimensional reconstructions of the brain of the mature dwarf male. *Symbion pandora*. (**A**) Dorsal view of the neuropil (red) through the transparent outline of the cuticle. Note the relative position of the neuropil inside the body. **(B-D)** Large scale view of neuropil (transparent red), one neuron and two bipolar sensory neurons. The neuron (light green) connects with the brain via a short process. The outermost sensory neuron (light violet) projects a process anteriorly to the anterior sensilla and another process into the neuropil. The innermost sensory neuron (cobalt blue) projects an axonal process inside the neuropil and also has complex processes directed ventrally (not represented) to connect with putative ventral sensilla. **(B)** Dorsal view. **(C)** Ventral view. **(D)** Posterolateral view.

In the brain of the mature male, the midregion of the neuropil is oval with a maximum diameter of ca. 4.7 μm and a minimum diameter of 2.5 μm (Figs. 3A, 4, 5, 6B,C). On each side of the brain, two bipolar sensory neurons project processes into the brain neuropil and into the periphery to connect to the anterior sensilla (stiff cilia; Figs. 4, 7A-D). Both the sensilla and the cytoplasm of the sensory neurons appear very electrondense (Fig. 4). In addition, two other bipolar sensory neurons, one on each side of the body, have peripheral processes that connect to ventral sensilla, as well as processes that project into the brain (Fig. 5, 7B-D). Contrary to the situation found in the younger male (see below), ventral longitudinal neurites are not manifest.

Besides the cerebral and medial secretory glands, a pair of relatively small anucleate gland cells (each gland with 2.8 μm in diameter) is located very close to the most posterior region of the ventral ciliated field (Figs. 3A,B, 6D-F). These glands are most probably the same as the ventral glands found in the young male (see below), though they appear larger in the mature male. The ventral glands are round and located very close to each other, which gives them a bilobed appearance (Fig. 6F). The vesicles inside these glands are spherical and electrondense, similar to the round vesicles of both the cerebral and the medial glands. The outlet of these ventral glands is not apparent. Two pairs of anucleate prostate glands are located in the mid-posterior region of the body close to the basis of the penial organ (Fig. 3C). One pair of prostate glands is located dorsal to the other (Fig. 3C, 6G-I). The more dorsal pair of prostate glands is drop-shaped and narrows ventrolaterally, while the more ventral pair has the shape of a horizontal S-curve. The outlets of all four glands are located lateral to the penial organ (Fig. 6I). The vesicles of the prostate glands are smaller and less electrondense than those found in the secretory glands of the male (Fig. 3A).

**Fig 8:**
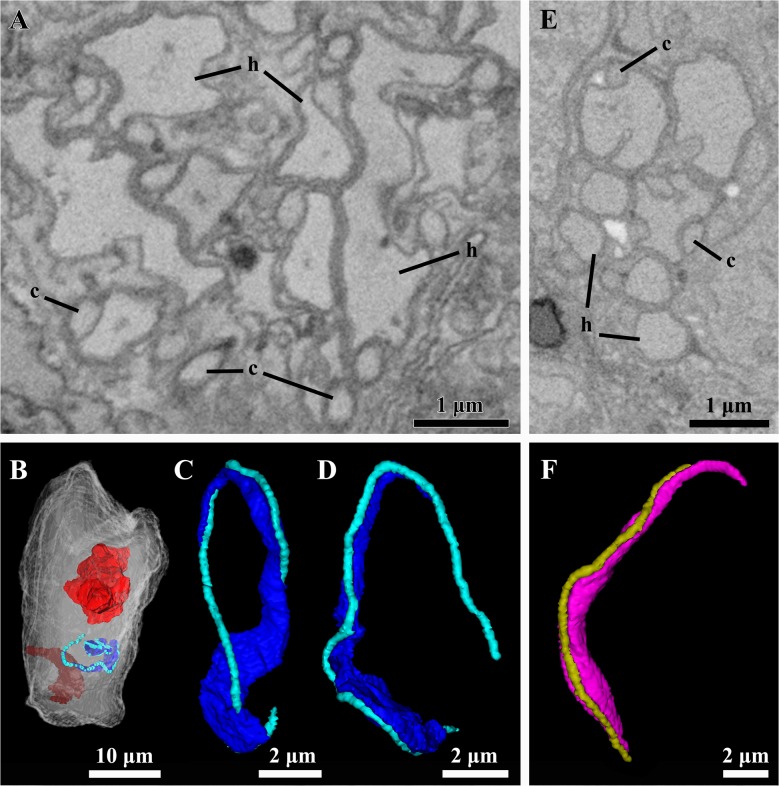
Ultrastructure and three-dimensional reconstructions of the sperm of the dwarf male. *Symbion pandora*. **(A)** Detail of the sperm cells of the mature dwarf male. (**B**) Lateral view of the neuropil (red), penis (grenat) and two sperm cells (heads are dark blue, while cilia are light blue) through the transparent outline of the cuticle of the mature male. Note the relative position of the sperm as compared to the penis. **(C-D)** Sperm cells of the mature dwarf male. Anterior region of the sperm cell is up. **(E)** Detail of the sperm cells of the young dwarf male. **(F)** Sperm cell of the young dwarf male (head is pink, while cilium is dark yellow). Anterior region of the sperm cell is up. Abbreviations: c cilium, h head of the sperm cell.

The penis of the mature male is characterized by a cuticle with an average thickness of 0.25 μm, which is continuous with body cuticle layer (Figs. 3A-D, 6C,I). The width of the penis is 3.3 μm at the base and 1.3 μm at the tip. As expected, all sperm cells contain a nucleus. Interestingly, a single sperm cell is located inside the penial organ (Fig. 3C). The testis is to a large extent occupied by 15 sperm cells, which are mainly filiform though posteriorly some of them are oval (Figs. 3A-C, 8A-D). The head of the sperm cell has a length of ca. 8.5–10.5 μm and a width of ca. 1.3–2 μm (Fig. 8A,C,D). The free part of the cilium is at least one third longer than the head, which is mainly occupied by the elongated nucleus. Other features typical of sperm cells such as mitochondria or an acrosome were not observed (Fig. 8A). The testis is not delimited by a membrane; its maximum diameter is 7.8 μm (Fig. 3A).

Although the myoanatomy of the mature adult is fully developed at this stage, nuclei related to the myocytes are not present. Similarly, the ventral and frontal ciliated fields are fully formed but the epithelium is basically reduced to a thin, anucleate layer, and solely three nuclei appear to belong to epithelial cells (two of these could also be mesenchymal). Contrary to the situation found in the young male, vacuolar structures are rarely found in the mature male body.

### The young dwarf male stage

A total of 208 somatic cells compose the body of the young dwarf male (see Table 1). Strikingly, and in marked contrast to the mature dwarf male, all of the cells in the young dwarf male are nucleated. The large majority of these cells, 114 (55%), are epithelial or mesenchymal. The brain (ganglia) includes 49 cells (23.6%), while muscles and secretory glands are composed of 32 (15.4%) and eight cells (3.85%), respectively. Moreover, only four cells (1.9%) appear to be related to the testis and only one cell (0.48%) appears to be associated with the prostate glands. Overall, the organs that typically characterize the anatomy of the cycliophoran dwarf male, e.g., muscles, brain, testis and glands, are already formed in the young male. The maximum length, height and width of the mature male is 30 μm, 14 μm and 34μm, respectively, which means a body volume of approximately14300 μm^3^ (see Table 2). Thus the immature dwarf male stage is approximately one third larger in body volume than the mature dwarf male stage. This size difference correlates well with the increase in total body volume that would be expected to result if all of the somatic cells retain their nuclei.

**Fig 9:**
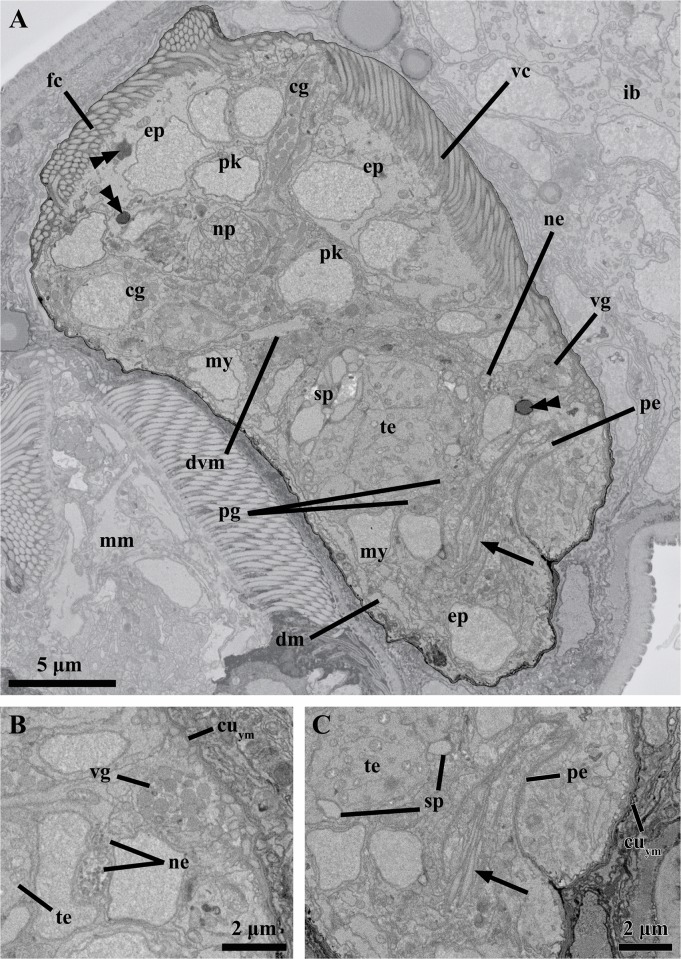
Anatomy of the young male inside the attached Prometheus larva of *Symbion pandora*. SEM-SBF micrographs, longitudinal section. (**A**) The young male is flanked dorsally by the mature male (mm) and ventrally by the internal bud (ib). The external morphology of the young male is characterized by frontal (fc) and ventral ciliated fields (vc). The anterior brain, including perikarya (pk) and neuropil (np), is well-developed. Note the cerebral glands (cg) surrounding the brain. The penis (pe) and the testis (te) characterize the posterior region of the body. Note as well the nuclei of myocytes (my) and epidermal cells (ep). (**B**) Close up of neurites (ne) spanning ventrally and a single ventral gland (vg). **(C)** Detail of the penis with a sperm cell inside. The arrow points to the cilium. Note that the sperm cells are located in the periphery of the testis. Abbreviations: cu_ym_ cuticle of the young male, dm dorsal muscle, double arrowheads vacuoles, dvm dorsoventral muscle, pg prostate glands, sp sperm cells, vm ventral muscle.

The nervous system of the young male, including the brain and a ventral pair of neurites, is fully developed. The mid region of the neuropil is more or less oval with a maximum diameter of ca. 3.8 μm and a minimum diameter of 2.8 μm (Fig. 9A). The two ventral neurites are found postero-ventral to the testis and appear to connect there (Fig. 9B). The brain is already surrounded by the three pairs of unicellular cerebral glands (Fig. 9A). These glands, as well as the pair of medial glands, are fully developed and possess individual outlets on the ventral side of the male body. In addition, a pair of small anucleate glands (each gland with ca. 2.3 μm in diameter) is located postero-ventral to the testis (and the ventral neurites), very close to the posterior end of the ventral ciliated field (Fig. 9B). The electrondense, round vesicles inside this pair of ventral glands appear similar to those found both in the cerebral and the medial glands. The four prostate glands are located between the testis and the penial organ (Fig. 9A). The vesicles of the prostate glands are smaller and less electrondense than those found in all other glands in the male body.

The penial organ is bounded by a thin cuticle, which is only 0.06 μm thick. The total width of this organ ranges from 2.8 μm at the base to 1.1 μm at the tip (Fig. 9A,C). Although a sperm cell is not clearly discernible inside the penial organ, at least one cilium is found inside this structure (Fig. 9C). The testis is located dorsally in the posterior region of the male body (Fig. 9A). It appears as a slightly electrondense amorphous mass rich in mitochondria. Moreover, 15 sperm cells occupy a peripheral position (Fig. 9A). These gametes are filiform with a head having ca. 8.5 μm in length and 0.9 μm in width (Fig. 8E,F); the free part of the cilium is slightly shorter than the head. The maximum diameter of the testis is 8.8 μm.

The body musculature of the young dwarf male is fully developed. Although myocyte nuclei are observed throughout the body, two thirds of their total number is concentrated in the posterior body region. Also scattered within the body volume are the mesenchymal and epithelial cells. The latter are multicilated cells which contribute to the well-developed ventral and frontal ciliated fields (Fig. 9A). These epithelial cells possess several mitochondria and usually have 1–2 bodies resembling vacuoles, which are electron dense in the periphery though relatively lucent in the interior (Fig. 9A). Vacuole-like bodies are also found in other cell types, e.g., in the nervous system.

### The internal bud-like stage

**Fig 10:**
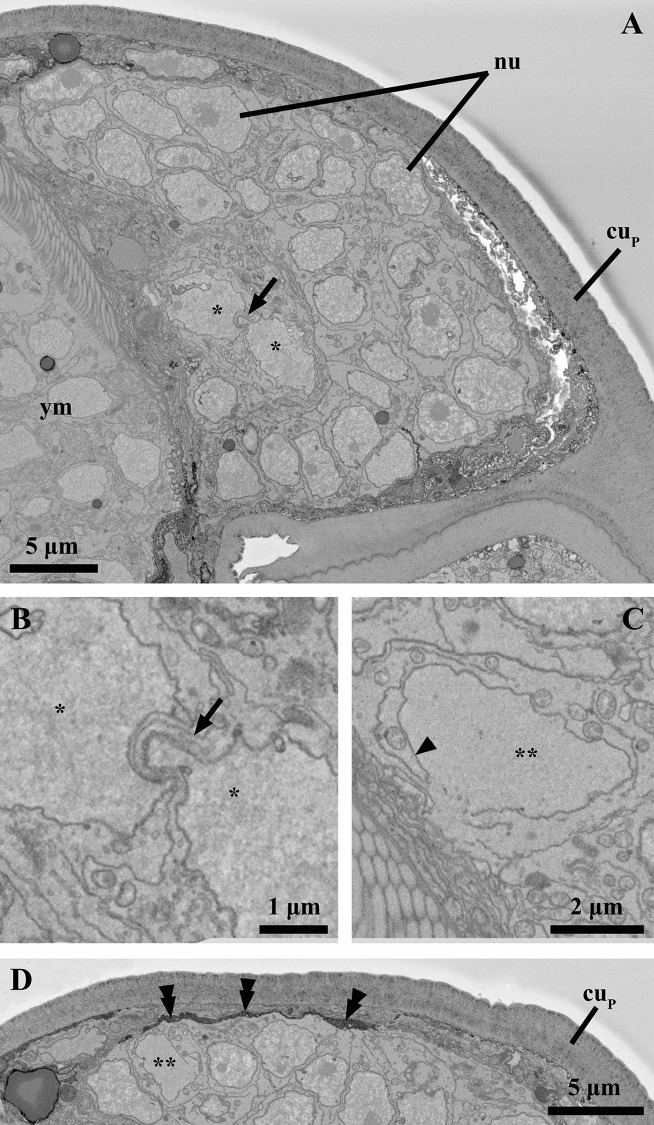
Ultrastructure of the internal bud inside the attached Prometheus larva of *Symbion pandora*. SEM-SBF micrographs, longitudinal section. (**A**) The internal bud is located anteriorly in the attached Prometheus larva, ventrally to the young male (ym). In this cluster, all cells look similar to each other except for two cells (marked with an asterisk) that appear to interact by means of pseudopodia (arrow). **(B)** Detail of the two cells interacting by pseudopodia. Note that the nuclear membrane of these cells is more irregular as compared to the other cells. **(C)** Detail of a cell (marked with two asterisks) characterized by a disrupted nuclear membrane (arrowhead). **(D)** The internal bud is outlined only by a basal lamina (double arrowheads). Note also the presence of a cell (marked with two asterisks) with a disrupted nuclear membrane. Abbreviations: cu_P_ cuticle of the attached Prometheus larva, nu nucleus.

The third, largely undifferentiated bud-like stage located at the most proximal region of the attached Prometheus larva consists of a cluster composed of 190 cells, all of which are nucleated and have several mitochondria in their cytoplasm (see Table 1, Fig. 10A). In these cells, the nucleus is large, containing one or two nucleoli, and occupies approximately 45–60% of the total cell volume. Morphologically, all cells look similar to each other, except for four (representing ca. 2%) located in the mid-distal region of the bud (Fig. 10A,B). These cells, found near the ventral region of the developing dwarf male, appear to interact with each other by means of pseudopodia. Moreover, the nuclear membrane of these cells is more irregular as compared to the other cells, and their cytoplasm is very rich in mitochondria. In addition, six cells (representing ca. 3%) are characterized by a disrupted nuclear membrane and, although several mitochondria are present in the cytoplasm, appear to undergo programmed cell death (Fig. 10C). With a length, height and width of 29 μm, 15 μm and 32 μm, respectively, the body volume of the bud-like stage is approximately13900 μm^3^ (see Table 2). Although the body volume and the total cell number at the bud-like stage is comparable to that of the young male stage, there are no signs of organogenesis at this bud-like stage. Neither myocytes, neural cells, secretory cells nor ciliated epidermis cells are present. Moreover, there is no indication that a cuticle is being formed; the largely undifferentiated cell cluster is outlined only by a basal lamina (Fig. 10D).

### The attached Prometheus larva

**Fig 11:**
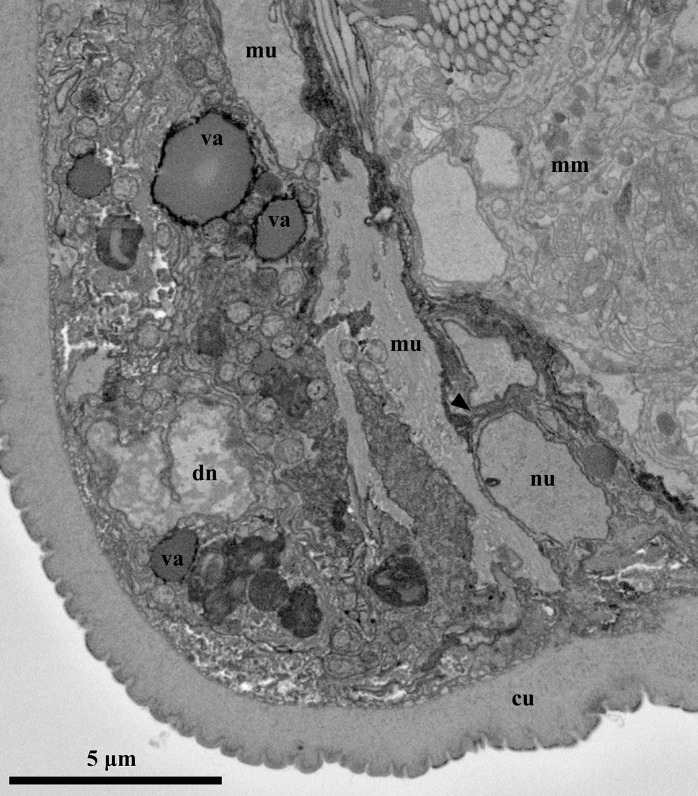
Ultrastructure of the attached Prometheus larva of *Symbion pandora*. SEM-SBF micrographs, longitudinal section. The musculature is the only organ system that remains in the settled Prometheus larva. Several muscle fibers (mu) surround the mature male (mm) and the myocyte still has nucleus (nu). The nucleus of an undetermined cell (epidermis?) is apparently undergoing a process of cell death (dn). Several vacuoles (va) are present throughout the whole body of the larva, which is delimited by a thick cuticle (cu).

With the exception of the larval musculature surrounding the dwarf males, no other organ systems are found inside the body of the attached Prometheus larva (Fig. 11). The larval body contains 56 cells and several of them appear to be undergoing a process of cell death (Fig. 11). In addition, the larval body is characterized by several large vacuoles that are spread all over the internal body volume (Fig. 11).

A cuticle layer that derives from the attached Prometheus larva delimits the postero-ventral region of the mature male such that the sculptured surface of the internal cuticle layer faces the male body (Fig. 3A,C). A similar situation is observed close to the postero-dorsal region of the male body, which is outlined by two layers of thick cuticle that are contiguous with the larval cuticle. In this case, however, the arrangement of the internal cuticle layer is different because its polygonal sculpture is facing the interior of the attached Prometheus larva. Moreover, the cuticle of the attached Prometheus larva outlining the most posterior region of the mature male appears discontinuous, and protrudes slightly from the rest of the larval cuticle. The cuticle in this region, as well as in the remaining regions of the larval body, is compact and without signs of fissures or opening.

## Discussion

The findings reported in this study are in accordance with a model for the development of the dwarf male with the following features. Initially, early proliferative processes within the Prometheus larva give rise to a largely undifferentiated cluster of approximately 200 cells. Subsequently, all of the cells in this cluster differentiate into specific cell types that form the major organs which are already present in the immature young male. Finally, a maturation process leads to the massive loss of cell nuclei in most of the organ systems resulting in a marked size reduction of the mature dwarf male. In the following, we discuss the major implications of these findings for our understanding of cycliophoran dwarf male organization and morphogenesis.

### The undifferentiated cell cluster: an early stage in dwarf male development

The presence of multiple internal buds and dwarf males co-existing simultaneously inside the same attached Prometheus larva is well documented in the two *Symbion* species hitherto described [9, 13, 10, 16]. It was actually useful to compare the earlier observations based on transmission electron microscopy (e.g., [9]) with our data set generated by SBF-SEM in order to identify structures such as cilia and muscle fibers that were not sufficiently stained. Indeed, the technique used in our study is highly dependent on staining procedures and electron density of the target structures. However, our ultrastructural analysis provides further insight into the organization of the internal bud as an early stage in dwarf male development. First, we find that the number of cells present in the internal bud stage is comparable to that of the young male. Second, we show that all of the cells comprising this stage are morphologically very similar to each other. Third, we find that none of the cells in this early stage of dwarf male development have undergone overt morphological differentiation into specific cell types.

The cycliophoran male seems thus to differentiate from an early stage that consists of a cluster of cells that are initially morphologically very similar and largely undifferentiated. At this early stage, differences between the approximately 200 cells are probably manifest at the molecular level in terms of differential gene expression, however, multipotency of these cells cannot be ruled out. How the cells composing the amorphous internal bud differentiate into organized structures (i.e., allocation of cells to the different germ layers) and when are the establishment of body axes occurring are yet open questions in dwarf male development.

### Dwarf male maturation involves body size reduction through massive loss of cell nuclei

The comparison between the two dwarf males investigated during this study, one young and the other mature, reveals new insight into the development of this cycliophoran life cycle stage. Our major novel findings concerning the young dwarf male stage are that it has already undergone organogenesis and established the morphological complexity that characterizes the cycliophoran male, and that all of its differentiated cells have nuclei. Thus, similar to the condition found in the mature male, the young male possesses well-developed nervous system, body musculature, reproductive system, glandular system and locomotory structures. Yet in striking contrast to the mature male, none of its cells have lost their nucleus indicating that a massive loss of nucleated cells occurs during the maturation process (see Tables 1 and 2). Equally striking is the fact that the young male is larger than the mature male; the internal body volume of the young male is 14300 μm^3^, while in the mature male is only 9000 μm^3^. Therefore, and contrary to the normal development of most animals, the cycliophoran dwarf male does not grow after organogenesis is concluded. On the contrary, the maturation of the young male into a mature male is a process that involves a reduction of about one third in the internal body volume.

The reduction in body size could be the result of a number of developmental processes including the absence of cellular growth and proliferation or the occurrence of programmed cell death and organelle loss. The fact that the cycliophoran male is confined to the body of the attached Prometheus larva which is shared with one or two other dwarf male stages, is also likely to be important for the remarkable miniaturization of the mature male body (see below). However, we posit that the major factor responsible for the reduction in body size in the mature stage as compared to the young stage is the extensive loss of nuclei in the majority of its somatic cells.

The elimination of nuclei in most of the somatic cells happens after organogenesis, when the organs are fully formed in the young male and genetic information in most somatic cells is not necessary for further development. Since the nucleus occupies a large part of the cell volume (e.g., nuclei represent 45–60% of the total volume of the internal bud), the process of anucleation appears to be a crucial step in the development of the miniaturized cycliophoran male. It is noteworthy, that the massive loss of nuclei during maturation appears to be most prominent in cells of the muscles and epidermis. In the cells of the mesenchyme cell lysis may also be occurring. In contrast, the cerebral and medial glands as well as the testis are only slightly smaller in the mature male than in the young male, while the ventral glands, neuropil, penis and sperm cells are slightly larger in the mature male than in the young male (see Table 2). These differences will only have minimal impact on the internal body volume. The difference between the number of cells composing the young male and the mature male explains the results obtained in earlier studies, in which 14 dwarf males of *Symbion pandora* were shown to possess an average of 52 somatic nuclei (this number ranged between 34 and 64 in 12 specimens) and solely two specimens possessed 78 and 84 somatic nuclei, respectively [14].

Although miniaturization seems to be a phenomenon widespread in Metazoa (animals), the process of cell anucleation is not commonly observed [19]. Recently anucleated neurons were reported to be present in the parasitic wasp genus *Megaphragma* [20]. In these very small insects (with body length of 170–200 μm), the adult brain and other ganglia of the central nervous system consist mainly of neuronal processes. Thus, the central nervous system of adult *Megaphragma* contains solely 339–372 nuclei, while other genus in the same family (i.e., Trichogrammatidae) possess about 37000 nucleated neurons only in the brain. The massive loss of nuclei is thought to take place after the formation of the adult nervous system, though before the hatching from the pupa, and significantly reduces the relative volume of the brain and other components of the nervous system. Although we are not aware of anucleated myocytes or anucleated ciliated epithelial cells reported in the literature, the example of *Megaphragma* provides a clear example of miniaturization of animals driven by anucleation of cells and might be comparable to what we observe in the cycliophoran dwarf male.

The extent to which the internal body volume of the attached Prometheus larva creates a physical limitation during the development of the dwarf male is not known. Recent work has shown that physical forces alone are sufficient to conduct the development of a complex structure. For example, investigations performed on chicken embryos demonstrate that lung morphogenesis is driven by apical constriction of the epithelium rather than by differential cell proliferation [21]. Whether or not comparable processes occur during the development of Cycliophora life stages will require more studies on the influence of physical forces on miniaturization in these remarkable animals.

### Comparative anatomy of the young and mature male stages

The anatomy of the mature male stage is different from that of the young male in various aspects. The most noticeable difference concerns the architecture of certain cell morphotypes that become anucleated, e.g., myocytes and epithelial cells, or are reduced to a much lower number of cells, which is the case of the mesenchymal cells. Nonetheless, some cell systems comprise exactly the same number of cells (e.g., secretory glands). As for the nervous system, the mature male appears to loose only a few cells as compared to the young male; in the specimens investigated here the difference is only of 15 cells.

Other differences exist concerning the reproductive system. For instance, the testis of the mature male is completely occupied by spermatozoa, while in the young male the gametes are distributed in the peryphery of an amorphous mass within the testis. The gametes found in both male stages are approximately of the same size and morphologically similar, i.e., filiform. However, in the mature male the free part of the cilium is longer than the head of the spermatozoon, while in the young male the cilium is slightly shorter than the gamete’s head. This could be indicative that (i) the gametes found in the young male are actually late spermatids yet undergoing development, and (ii) the amorphous mass within the testis is a supportive tissue supplying nutrients to these late spermatids. Because the number of spermatozoa is similar both in the mature male and in the young male, it is likely that no further gametes will be produced in the testis of the young male. Interestingly, filiform sperm cells with elongated nucleus are also found in Entoprocta [22, 23], a phylum that is traditionally regarded as the sister group of Cycliophora (as a review see [24]). However, before a comparative study of gametes can be performed, a thorough investigation by transmission electron microscopy is necessary to clarify the presence of a mitochondrion and an acrosome in the cycliophoran sperm as suggested earlier [9].

Concerning further aspects of the reproductive system, the penial cuticle of the mature male looks thicker than that of the young male, and this appears to be an indication for maturity in this life cycle stage. The presence of a sperm cell inside the penial organ of the mature male, as well as in the young male, provides support for the view of the penis is indeed a true copulatory organ and not only a mere anchoring cirrus [9, 15]. Although the tip of the penis is not open, its width of ca. 1.3 μm is approximately the same size as the maximum diameter of the sperm cells. Therefore, the rupture of the cuticle at the tip of the penis could enable the exit of the sperm cells during a putative copulation with the cycliophoran female. However, the process through which the tip of the penis is opened remains obscure.

In general, the anatomy and external morphology of the mature dwarf male described here are in accordance with earlier descriptions of this cycliophoran adult stage [9, 10, 13, 15, 17]. However, the pair of secretory glands located ventrally in the posterior region of the male body is described here for the first time. The spherical vesicles contained in these ventral glands appear similar to the round vesicles found in, e.g., the anterior glands of the chordoid larva and the cycliophoran female [11, 12]. Since in both cases these vesicles contain mucus substances, we interpret the single ventral gland as a mucus gland which is involved in the crawling of the dwarf male. In addition, our observations on the sensory neurons that project a peripheral process ventrally support the presence of ventral sensilla in the cycliophoran dwarf male. This cellular process connects to electrondense cilia, which are very similar to those located anteriorly. Because only anterior and lateral sensilla have been described so far in cycliophoran males, more studies on their external morphology are desirable to confirm the presence of ventral sensilla.

### Role of the Prometheus larva

As the only organ system observed in the attached Prometheus larva, the several muscle fibers that encircle the two dwarf males appear to have a protective role corroborating earlier assumptions [10, 13]. Furthermore, it is interesting to observe that the larval musculature does not encircle the internal bud and, hence, does not protect this undifferentiated cluster of cells.

The finding of short layers of sculptured cuticle inside the attached Prometheus larva, which partially surround the mature dwarf male, might illuminate the process used by this adult stage to escape from the larval body. Because the cuticle of the attached Prometheus larva investigated here is compact all over the body and without signs of having an open (e.g., a putative pore as described earlier [3, 9]), it is probable that the mature dwarf male escapes only after the rupture of the larval cuticle. We regard the region of the larval cuticle outlining the most posterior region of the mature male, which appears discontinuous and slightly protruding from the rest of the cuticle layer, as a location where the rupture could eventually occur. After the fracture of the larval cuticle during the escape of the male, this structure could subsequently be regenerated using the internal layer of sculptured cuticle that partially surrounds the ventral region of the male. The length and orientation of this short cuticle layer is, indeed, adequate to fill a putative gap in the larval cuticle generated by the exit of the male. Nonetheless, further studies on this subject are desirable to fully understand this aspect of the cycliophoran life cycle.

## Supporting Information

S1 Video Anatomy of the attached Prometheus larva containing inside a mature dwarf male, a young dwarf male and a bud-like stage. *Symbion pandora*. A movie sequence through the stack of SEM-SBF micrographs (longitudinal sections) investigated in this study.
(AVI)
